# Der Wandel von Vertrauen in eine digitale Identität? – Einblicke in eine Nutzerstudie

**DOI:** 10.1365/s40702-023-00951-7

**Published:** 2023-03-02

**Authors:** Sandra Kostic, Maija Poikela

**Affiliations:** 1 Fraunhofer-Institut für Angewandte und Integrierte Sicherheit AISEC, Gaching bei München, Deutschland; 2 Berliner Institut für Gesundheitsforschung in der Charité, Charité – Universitätsmedizin Berlin, Berlin, Deutschland

**Keywords:** Digitale Identität, Identity Wallet, Nutzerstudie, Vertrauen, Usability, Benutzbare Sicherheit, Digital Identity, Identity Wallet, User Study, Trust, Usability, Usable Security

## Abstract

Ausweisdokumente ermögliche es Personen vor Ort eindeutig zu identifizieren. Um bestimmte online Dienste wahrzunehmen zu können, bedarf es auch einer Identifikation im Internet. Hierfür wird eine digitale Identität benötigt.

Dieser Beitrag stellt die Ergebnisse von zwei Studien mit Nutzenden (mit jeweils 16 und 12 Teilnehmenden) vor, die auf einem neu entwickelten Konzept einer sogenannten Identity Wallet basieren. Dieses Konzept veranschaulicht, wie Nutzende selbstständig diverse digitale Identitäten, sowohl hoheitliche wie der Personalausweis als auch nicht-hoheitliche wie der Bibliotheksausweis, in einer einzigen App speichern. Somit ist es den Nutzenden möglich ihre Identität mit einer einzigen App bei Dienstanbietern mit unterschiedlichsten Anforderungen an das Ausweisdokument nachzuweisen. Neben der Speicherung von Ausweisen oder auch Nachweisdokumenten, zeigt dieses Identity Wallet Konzept ebenso die Option auf Schlüssel (Fahrzeugschlüssel, Hotelzimmer, etc.) in derselben App zu hinterlegen.

Das Konzept wurde 2020 ausgearbeitet und mit 16 Studienteilnehmenden getestet, um nicht nur die Einsatzbereitschaft der Nutzenden, sondern auch das Vertrauen in solch ein Konzept zu evaluieren. Die Teilnehmenden waren offen für den Einsatz der Wallet und vom Konzept überzeugt. In Bezug auf das Thema Vertrauen wiesen die Ergebnisse der Studie daraufhin, dass der Anbieter der Wallet Anwendung Einfluss darauf nimmt, inwieweit Nutzende beschließen, der Anwendung zu vertrauen. Etwa die Hälfte der Teilnehmenden bevorzugten den Staat als Betreiber der Wallet, während die übrigen Teilnehmenden ein privates Unternehmen präferierten.

Ein überarbeitetes Konzept der Identity Wallet wurde 2022 erneut mit 12 Studienteilnehmenden getestet. Auch hier wurde die Frage der Einsatzbereitschaft der Nutzenden sowie des Vertrauens evaluiert. Die Studienteilnehmenden zeigten weiterhin eine große Bereitschaft zum Einsatz der Wallet. Allerdings veränderten sich die Ergebnisse zum Thema Vertrauen. Sie weisen darauf hin, dass nur eine Person ein privates Unternehmen als Betreiber der Wallet bevorzugt, während die übrigen Teilnehmenden den Staat favorisieren.

**Zusatzmaterial online:**

Zusätzliche Informationen sind in der Online-Version dieses Artikels (10.1365/s40702-023-00951-7) enthalten.

## Abstract (en)

ID documents make it possible to identify people uniquely on site. In order to use certain online services, identification is also required on the Internet. This requires a digital identity.

This paper presents the results of two studies with users (16 and 12 participants each) based on a newly developed concept of an identity wallet. This concept illustrates how users can independently store various digital identities, both sovereign ones like the ID card and non-sovereign ones like the library card, in a single app. This makes it possible for users to prove their identity with a single app to service providers with a wide range of requirements for the ID document. In addition to storing IDs or verification documents, this identity wallet concept also shows the option to store keys (car keys, hotel rooms, etc.) in the same app.

The concept was elaborated in 2020 and tested with 16 study participants to evaluate not only the readiness of the users but also the trust in such a concept. The participants were open to using the wallet and were convinced by the concept. In terms of trust, the results of the study indicated that the provider of the wallet application influences the extent to which users decide to trust the application. About half of the participants preferred the government as the operator of the wallet, while the remaining participants preferred a private company.

A revised concept of the Identity Wallet was tested again in 2022 with 12 study participants. Again, the question of user readiness and trust was evaluated. The study participants continued to show a high level of willingness to use the Wallet. However, the results on trust changed. They indicate that only one person prefers a private company to operate the Wallet, while the remaining participants favor the state.

## Einführung

Anhand vom Vornamen, dem Nachnamen und dem Geburtsdatum ist es möglich eine Person eindeutig zu identifizieren (Sweeney 2000). Sogenannte digitale Identitäten werden benötigt, um Personen online zu identifizieren. Dienste wie Facebook und Google bieten derzeit bereits Beispiele einer digitalen Identität an (Login with Facebook^1^ oder Google^2^) (Micallef et al. 2018). Nutzenden können bei diesen Diensten einen Account mit ihren persönlichen Daten erstellen und dieses Konto – ihre von Facebook oder Google ausgestellte digitale Identität nutzen – um von anderen Diensten identifiziert zu werden.

Es gibt jedoch zwei Probleme mit diesen digitalen Identitäten:

1. Diese von Google oder Facebook bereitgestellt digitale Identität kann nicht für Onlinedienste genutzt werden, welche eine Identifizierung mit einem verifizierten hoheitlichen Dokument anfordern.
2. Diese Identitäten werden bei den Diensten gespeichert. Die Nutzenden und damit die Eigentümer der Daten sind diesen Diensten ausgesetzt, welche entscheiden, was sie mit den Daten machen und wie sie verarbeitet werden (Krasnova et al. 2014; Scott et al. 2016; Karegar et al. 2018)

Mit der AusweisApp2^3^ gibt es in Deutschland bereits eine Lösung, welche eine digitale Identifizierung mit dem deutschen Personalausweis und damit mit einem hoheitlichen Dokument ermöglicht (Noack und Kubicek 2010). Anwendungen, wie die AusweisApp2, stellen jedoch hohe Anforderungen an die Nutzenden, die erfüllt sein müssen, damit der Personalausweis in der digitalen Welt eingesetzt werden kann. Das schließt unter anderem die Aktivierung der sogenannten Onlineausweisfunktion des Personalausweises (Bundesministerium des Inneren und für Heimat 2022a) sowie den Besitz von bestimmter Hardware ein, um den Personalausweis digital auslesen zu können (z. B. ein kompatibles Smartphone mit NFC-Schnittstelle als Kartenleser) (AusweisApp2 2022). Zudem gibt es Hinweise, dass diese Verfahren nicht immer benutzerfreundlich sind, wodurch ihr Einsatz erschwert wird (Asheuer et al. 2013; Willomitzer, Heinemann and Margraf, 2016).

Das Konzept von Self-Sovereign-Identity (SSI) liefert eine Alternative, womit ebenso digitale Identitäten erstellt werden können. Für die Erstellung dieser müssten jedoch weniger hohe Anforderungen erfüllt werden. Hinter diesem Konzept verbirgt sich ein Ansatz bei den beispielsweise die Nutzenden nicht nur eigenständig die digitale Identität erzeugen, sondern diese auch vollständig kontrollieren können, ohne die Notwendigkeit eines Vermittlers oder einer zentralen Partei (Der et al. 2017; Preukschat und Reed 2021). Die Nutzenden beschließen eigenständig, welche Daten sie teilen wollen.

Die Schwierigkeit dieses Ansatzes liegt jedoch darin, dass die digitale Identität eine Quelle benötigt, die diese Identität als verifiziert belegt. Damit ist es zwar theoretisch möglich Ausweisdokumente, wie den Personalausweis zu digitalisieren, doch mit dem fehlenden Status „verifiziert“, kann nicht das gleiche hohe Vertrauensniveau wie z. B. in der AusweisApp2 bereitgestellt werden (Fromm et al. 2013; Amtsblatt der Europäischen Union 2014), was dazu führen kann, dass die Self-Sovereign Identity nur von wenigen Dienstleistern als Identifikationsmittel akzeptiert wird (Cuijpers und Schroers 2014; Der et al. 2017). Darüber hinaus gibt es Hinweise darauf, dass die Apps, welche die SSI Technologie bereitstellen, große Herausforderungen in Bezug auf die Benutzerfreundlichkeit mit sich bringen (Khayretdinova et al. 2022; Sartor et al. 2022).

In Anbetracht der drei genannten Konzepte zu digitalen Identitäten lassen sich folgenden Anforderungen zusammenfassen:

1. Die digitale Identität sollte selbsterklärend und mit geringen Anforderungen erstellt werden können.
2. Die Nutzenden müssen in der Lage sein die digitale Identität sowie ihre Daten zu kontrollieren.
3. Die digitale Identität soll von möglichst vielen Dienstanbietern als Identifikationsmittel akzeptiert werden.

Um diese Anforderungen zu erfüllen, wurde ein Konzept einer digitalen Identity Wallet^4^ (eine digitale Brieftasche) erarbeitet (Schaarschmidt et al. 2022). Sie ermöglicht es verschiedene digitale Identitäten und Nachweisdokumente aus unterschiedlichen Quellen mit unterschiedlichen Vertrauensniveaus in einer App zu speichern. Auf diese Weise können die Nutzenden gleichzeitig Identitäten nutzen, die höhere Anforderungen bei der Erstellung stellen, aber eine große Bandbreite bei der Nutzung haben, sowie Identitäten, die niedrige Anforderungen haben, aber in für die Nutzenden relevanten Anwendungsfällen eingesetzt werden können.

Dieses Konzept veranschaulichte unter anderem die Digitalisierung des Personalausweises, die Onlineidentifizierung, das Speichern des Bibliotheksausweises sowie das Speichern von Schlüsseln (Hotelzimmer und Auto). Damit soll ein kleiner Blick in die Breite der Anwendungsfälle geliefert werden, da dieses Konzept der Wallet sowohl im staatlichen als auch im Privatsektor eigesetzt werden kann.

Dieses Konzept wurde basierend auf einer Befragung im Rahmen einer Fokusgruppe entwickelt.

Um dieses Konzept zu evaluieren, wurden folgende drei Forschungsfragen (FF) untersucht.

1. FF1: Wie verständlich ist das Konzept der digitalen Identity Wallet und wollen Nutzende dieses einsetzen?
2. FF2: Inwieweit sind sich die Nutzenden bewusst, dass ihre gespeicherte digitale Identität aus einem hoheitlichen Dokument stammt?
3. FF3: Welche Faktoren beeinflussen die Wahrnehmung der Kontrolle über die Daten?

Zur Beantwortung dieser Forschungsfragen, wurde 2020 eine Studie mit 16 Teilnehmenden durchgeführt (siehe Abschn. 4).

Alle Teilnehmer waren in der Lage, erfolgreich eine Identität zu erstellen und verstanden den Identifikationsprozess mit dem Personalausweis. Die Teilnehmer zeigten ebenso eine große Bereitschaft die Wallet einsetzen zu wollen, da sie unter anderem den Eindruck vermittelt bekamen stets die Kontrolle über ihre Daten zu besitzen.

Da im Rahmen der Wallet personenbezogene Daten gespeichert werden, war es wesentlich für die Studie mit den Nutzenden nicht nur zu evaluieren, ob sie den Einsatz der Wallet verstehen, sondern inwieweit sie auch dieser vertrauen.

Die Ergebnisse legen nahe, dass der Wallet-Betreiber eine wichtige Rolle dabei spielt, ob die Studienteilnehmenden darauf vertrauen, dass ihre Daten in der Wallet verantwortungsvoll gehandhabt werden. Etwa die Hälfte der Studienteilnehmenden sahen den Staat als die einzige akzeptable Option für den Betreiber der Wallet, während die übrigen Teilnehmenden ein privates Unternehmen bevorzugten.

Im Jahr 2022 wurde ein überarbeitetes Konzept einer Identity Wallet erneut im Rahmen einer Studie mit Nutzenden getestet (siehe Abschn. 7). Hierbei wurden 12 Personen befragt. Der Fokus dieser Studie lag, statt auf der Erstellung und Nutzung des digitalen Personalausweises auf die Digitalisierung des mobilen Führerscheins. Auch in dieser Studie sollte die Bereitschaft zum Einsatz der Wallet sowie das Vertrauen untersucht werden.

Das Ergebnis der Studie im Jahr 2022 zeigt auf, dass die Teilnehmenden der Studie die Wallet weiterhin verwenden wollen. Hinsichtlich des Vertrauens in die Wallet, hat sich die Einstellung der Nutzenden zum Betreibermodell geändert. Die Ergebnisse zeigen nun auf, dass nur eine Person ein privates Unternehmen als Betreiber der Wallet bevorzugt, wohingegen die übrigen Teilnehmenden sich den Staat als Betreiber wünschen.

**Abb. 1:**
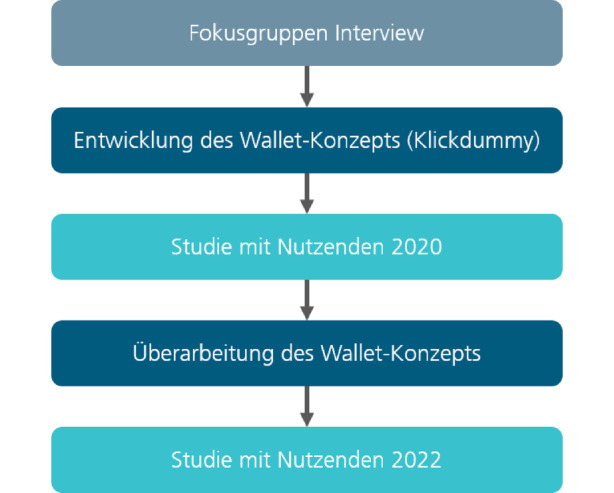
Phasen der Untersuchung

Abb. 1 fasst zusammen, welche Phasen im Rahmen dieses Beitrags durchlaufen wurden.

Die Ergebnisse der Studie aus 2022 werden erneut genutzt, um in zukünftigen Arbeiten ein überarbeitetes Konzept einer Wallet zu entwickeln und die Integration weiterer Anwendungsfälle vorzusehen.

Zur Veranschaulichung der Inhalte in diesem Beitrag wird in Abschn. 3 zunächst das Wallet-Konzept vorgestellt. Es beinhaltet die Beschreibung eines Funktionsumfangs und präsentiert das Konzept anhand von Low Fidelity Mockups (Virzi et al. 1996). In Abschn. 4 wird die erste Nutzerstudie durchgeführt im Jahr 2020 zur Evaluierung des Konzepts vorgestellt. Darauf folgt in Abschn. 5 die Auflistung der Ergebnisse der Nutzerstudie aus 2020. Abschn. 6 beinhaltet die Beschreibung eines überarbeiteten Konzepts der Wallet inklusive eines erweiterten Funktionsumfangs sowie überarbeiteter High Fidelity Mockups (Virzi et al. 1996). In Abschn. 7 wird die Nutzerstudie durchgeführt im Jahr 2022 vorgestellt, gefolgt von den Ergebnissen der Studie aufbereitet in Abschn. 8. Anschließend werden Ergebnisse der Nutzerstudien aus 2020 und 2022 in Abschn. 9 miteinander verglichen. Der Beitrag schließt mit den Einschränkungen in Abschn. 10 sowie mit der Zusammenfassung und dem Ausblick in Abschn. 11.

## Verwandte Arbeiten

Heutige Wallets befinden sich aktuell im starken Entwicklungsprozess und zeigen Herausforderungen in der Benutzerführung (Khayretdinova et al. 2022; Korir et al. 2022; Sartor et al. 2022). So gibt es grundsätzlich Verbesserungsbedarf in der Erklärung der Technologie der Wallets und ihrer Vorteile den Nutzenden gegenüber (Khayretdinova et al. 2022; Sartor et al. 2022). Damit soll es zu großen Problemen und zur Beeinträchtigung der Technologie führen. Zudem weisen die Wallets unzureichenden Beschreibungen der Terminologien vor (Khayretdinova et al. 2022) und Korir et al. wiesen darauf hin, dass Nutzenden Bedenken äußern zu viele Daten mit ihrer Wallet zu teilen (Korir et al. 2022). Grundsätzlich wurden von Khayretdinova et al. angemerkt, dass die Funktionen in den untersuchten Wallets nicht selbsterklärend von den Nutzenden verstanden wurden (Khayretdinova et al. 2022). Außerdem mangelte es an einer einfachen Handhabung sowie guten Benutzerführung.

## Wallet-Konzept

Das Konzept dieser Wallet besteht darin, die einfache und sichere Speicherung von Identitäten, Nachweisen und Schlüsseln in einer Smartphone-Anwendung zu ermöglichen. Diese App unterstützt nicht nur die Erstellung einer digitalen Identität, welche nur für den Besitzenden der Wallet-App angelegt werden kann, sondern auch die Speicherung von Identitäten, die von anderen Ausstellern bereitgestellt werden (wie z. B. Bibliotheksausweis, Studentenausweis, Mitarbeiterausweis). Ziel ist es, den Nutzenden die Entscheidung zu überlassen, welche Identität sie in der Wallet speichern wollen, und ihnen eine breite Palette von Möglichkeiten anzubieten.

Die Besitzenden der Wallet sollen jederzeit die Kontrolle über die gespeicherten Daten haben und selbst entscheiden, welche genauen Daten für den gewünschten Zweck an einen Dienst gesendet werden sollen.

Da die Wallet persönliche Daten speichert und potenzielle Nutzende der Wallet vertrauen sollen, wurden mit Hilfe einer Fokusgruppe (bestehend aus 6 Personen) Anforderungen an den Funktionsumfang der Wallet gesammelt (siehe Abschn. 3.1).

### Fokusgruppen

Um bei der Entwicklung des Wallet-Konzepts gleich zum Anfang die Nutzenden einzubeziehen und ihre Anforderungen hinsichtlich der Sicherheit und Privatsphäre zu ermitteln, wurde im Juli 2022 ein Workshop mit einer Fokusgruppen über 4 h mit 6 Personen durchgeführt. Für die Interviews wurden Personen über den Service Testing Time^5^ akquiriert. Die Teilnehmenden der Studie waren gleichverteilt über Alter und Geschlecht vertreten und haben eine Aufwandsentschädigung erhalten. Dabei wurden Personen ausgeschlossen, welche Experten auf dem Gebiet Security, UX Design oder Usability waren. Das Ziel dieser Fokusgruppe war es unteranderen Funktionen für das Wallet-Konzept zu ermitteln. Hierfür haben die Studienteilnehmenden eine kurze Einleitung in die Thematik von digitalen Identitäten und ihren Einsatz erhalten.

**Abb. 2:**
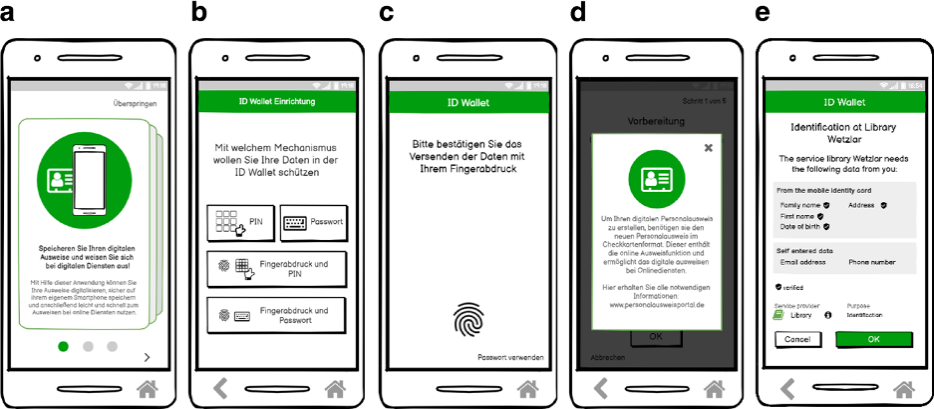
Umgesetzte Anforderungen aus der Fokusgruppe in das Wallet-Konzept*. ***a** Einleitung in die Wallet, **b** Einstellungsmenü zur Festlegung des Entsperrmechanismus, **c** Datenweitergabe nur mit zusätzlicher Zustimmung des Nutzenden, **d** Hilfefenster für weitere Informationen, **e** Übersicht über angefragte Daten

Folgende Liste fasst die Anforderungen der Teilnehmenden aus der Fokusgruppe zusammen. Diese wurden in das Wallet-Konzept umgesetzt (siehe Abb. 2):

- Bereitstellung einer Einleitung, welche im Rahmen des Onboardingprozesses den Funktionsumfang der Wallet vorstellt (siehe Abb. 2a).
- Das Festlegen eines Entsperrmechanismus, sodass der Zugriff auf die gespeicherten Daten geschützt ist (siehe Abb. 2b).
- Nur mit einer zusätzlichen Zustimmung werden Daten an einen Dienst weitergesendet (siehe Abb. 2c).
- Hilfemenüs sollen weitere Informationen zum Einsatz der Wallet bereitstellen (siehe Abb. 2d).
- Dem Nutzenden soll übersichtlich dargestellt werden, welche Daten vom Dienst erfragt werden, bevor sie versendet werden (siehe Abb. 2e).

### Funktionen der Wallet

Nach der Durchführung der Fokusgruppen wurde mit der Umsetzung des Wallet-Konzepts begonnen. Die Abb. 2, 3 und 4 zeigen die Screens des umgesetzten interaktiven Low Fidelity Klick Dummy.

**Abb. 3:**
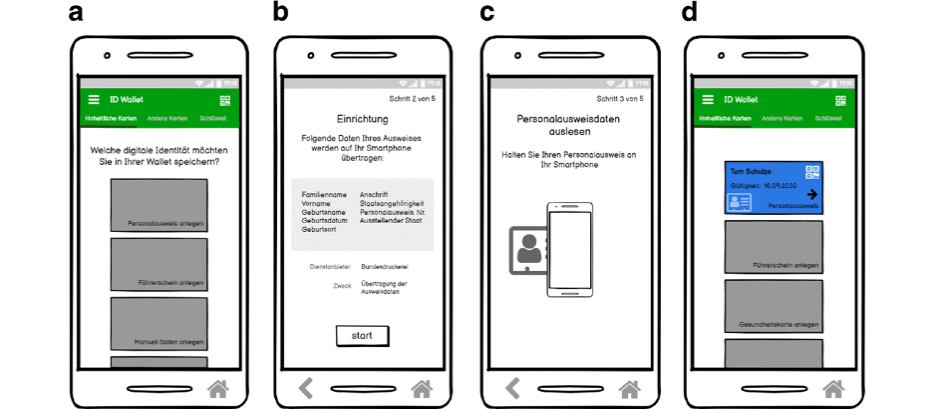
Digitalisierung des Personalausweises. **a** Übersicht der Identitäten, welche gespeichert werden können, **b** Einrichtungsbildschirm mit Datenübersicht, **c** Speichern der Identität durch Scannen des Personalausweises, **d** Gespeicherter Personalausweis auf dem Smartphone

**Abb. 4:**
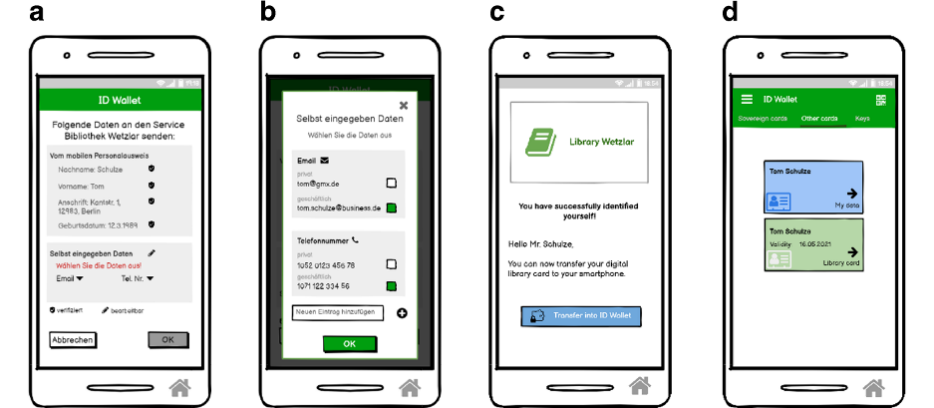
Identifikation und Übertragung des Bibliotheksausweises in die Wallet. **a** Übersicht über versendete Daten, **b** Übersicht über versendete Daten, **c** Erfolgreiche Identifikation und Anfrage zur Übertragung des digitalen Büchereiausweises, **d** Gespeicherter digitaler Büchereiausweis in der Wallet

Das Konzept der Wallet umfasst folgende Kernfunktionen. Sie orientieren sich an den Ergebnissen der Fokusgruppe:

1. Festlegen eines Schutzmechanismus
2. Digitalisierung des nationalen Personalausweises (siehe Abb. 3)
3. Identifizierung mit den in der Wallet gespeicherten Identitäten
4. Übertragung von Identitäten in die Wallet von anderen Herausgebern (am Beispiel Bibliothek) (siehe Abb. 4)
5. Speicherung von Schlüsseln
6. Digitalisierung des Führerscheins mit dem Service der Führerscheinstelle

Einige der Funktionen werden im Folgenden näher beschrieben.

#### Festlegen eines Schutzmechanismus

Um den unbefugten Zugriff auf die Wallet zu verhindern, können Nutzende entweder den bereits etablierten Entsperrmechanismus des Smartphones verwenden oder einen neuen Schutzmechanismus (PIN, Passwort, Fingerabdruck), welcher nur für die Wallet gilt, einstellen (siehe Abb. 2b).

#### Digitalisierung des nationalen Personalausweises

Um den Personalausweis als Ausweisdokument zu verwenden, kann der Nutzende den Personalausweis ausschließlich über die Wallet-App digitalisieren. Dieser digitale Personalausweis kann nur für den Besitzenden der Wallet erstellt werden. Dazu wird die Einrichtungskarte des Personalausweises angeklickt, um den Prozess zu starten (siehe Abb. 3a). Nach Bestätigung der Infoseite zur Beschreibung der benötigten Informationen zur Einrichtung des digitalen Personalausweises (siehe Abb. 3b), werden die Nutzenden aufgefordert ihren Personalausweis mit Hilfe der NFC-Schnittstelle des Smartphones auszulesen (siehe Abb. 3c) und die Personalausweis-PIN einzugeben. Hierbei besteht die Idee des Konzepts den Ausweis mit Hilfe des Secure Element^6^ ausschließlich auf dem Smartphone sicher zu speichern (Rohilla 2015; Schwan und Ohlendorf 2019). Somit ist es möglich den Ausweis als hoheitliches und verifiziertes Dokument einzusetzen. Am Ende des Vorgangs wird der Personalausweis als in der Wallet gespeicherte Karte angezeigt (siehe Abb. 3d).

Dabei sei anzumerken, dass bei einer Umsetzung dieses Ansatzes der Einsatz von Secure Elements mit gewissen Limitierungen einhergeht, da derzeit nur eine geringe Anzahl von Endgeräten kompatibel zur Verwendung eines Secure Elements sind (Schütte 2014; Schaufenster Sichere Digitale Identitäten 2022).

#### Identifizierung mit den in der Wallet gespeicherten Identitäten

Nachdem Ausweisdokumente in der Wallet gespeichert wurden, können sie sowohl für die Online-Identifizierung als auch für die Identifizierung vor Ort mit einem QR-Code verwendet werden. Die Wallet kann mit anderen Diensten, die eine Identifikation erfordern, kommunizieren. Der Nutzende erhält vorab eine Übersicht, welche Daten angefordert werden (siehe Abb. 2e) sowie welche konkreten Daten versendet werden (siehe Abb. 4a). Diese können aktiv im Prozess der Anfrage ausgewählt werden (z. B. welche Emailadresse) (siehe Abb. 4b).

Der Nutzende ist in der Lage während der eingehenden Anfrage weitere Details zum anfragen Dienst angezeigt zu bekommen (z. B. ob ein gültiges Berechtigungszertifikat für diese Anfrage vorliegt) und versendet die eigenen Daten nur mit einer zusätzlichen Zustimmung (z. B. durch Bestätigung mit einer PIN oder einem Fingerabdruck) (siehe Abb. 2c).

#### Übertragung von Identitäten in die Wallet von anderen Herausgebern

Nach erfolgreicher Identifizierung können die von einem Dienstanbieter bereitgestellten Identitäten über einen Link^7^ aus der App des Anbieters oder durch Scannen eines QR-Codes^8^ in die Wallet übertragen werden (siehe Abb. 4c und d für den Bibliotheksausweis). Diese Identitäten können wiederum als Identifikationsmittel verwendet werden, sowohl online als auch vor Ort.

## Erste Nutzerstudie (2020)

Um nicht nur zu untersuchen, ob das Konzept von Nutzenden verstanden wird und ob sie bereit wären dieses nutzen zu wollen, sondern auch im Besonderen inwieweit sie diesem Konzept einer Identity Wallet vertrauen, wurde eine Studie mit insgesamt 16 Nutzenden im Herbst 2020 durchgeführt.

Die Teilnehmenden im Alter zwischen 18 und 56 Jahren wurden über die gebührenpflichtige Plattform Testing Time akquiriert. Es wurden, wie auch bei den Fokusgruppen (siehe Abschn. 3.1), Personen ausgeschlossen, welche sich selbst als Experten in den Bereichen Sicherheit, UX-Design und Usability eigeordnet hatten. Darüber hinaus wurde beachtet, dass auch in dieser Studie eine Gleichverteilung hinsichtlich des Geschlechts und des Alters vorhanden ist. Jede Sitzung pro Studie wurde für 90 min angesetzt.

Aufgrund der COVID-19-Pandemie wurde die Studie in digitaler Form mit einem Videokonferenz-Tool durchgeführt. Der digital aufbereitete interaktive Prototyp wurde den Teilnehmenden über einen Link zur Verfügung gestellt, und sie wurden gebeten, ihren Bildschirm in einer Online-Sitzung freizugeben, damit sie bei der Bedienung des Prototyps beobachtet werden konnten.

Die Studienteilnehmenden erhielten Aufgaben, die sie mit Hilfe des Prototyps lösen sollten. Nach jeder Aufgabe wurden sie befragt, um weitere Einzelheiten über ihre Wahrnehmung und ihr Verständnis des Prototyps zu erfahren.

Die Aufgaben waren die folgenden:

1. Einrichten der Wallet und Festlegen einer Wallet-PIN.
2. Übertragung des Personalausweises in das Smartphone.
3. Eine Online-Identifizierung mit dem auf dem Smartphone gespeicherten digitalen Personalausweis.
4. Übertragung des digitalen Bibliotheksausweises von der digitalen Bibliothek auf die Wallet (Kommunikation von App zu App).
5. Die Erstellung eines digitalen Führerscheins (Kommunikation von Web zu App).
6. Die Speicherung eines Fahrzeugschlüssels für ein gemietetes Fahrzeug.

Während dieser Aufgaben wurde die Methode des Think Aloud verwendet (van Someren et al. 1994), die es einem ermöglicht, nicht nur die Handlungen der Teilnehmenden während der Studie zu beobachten, sondern auch ihre Gedanken, Annahmen und Kommentare zu notieren. Anschließend wurde ein abschließendes Interview geführt, um den Gesamteindruck der Teilnehmenden zu ermitteln.

## Ergebnisse

### Verständlichkeit und Akzeptanz

Alle Teilnehmenden (16 von 16) waren in der Lage, erfolgreich eine digitale Identität zu erstellen und diese für die digitale Identifizierung zu nutzen. Aufgrund der durchgeführten online Studie bedingt durch die Pandemie, war es jedoch nicht möglich eine Aussage über eine konkrete Erfolgsquote zu treffen. Dafür ist es erforderlich, dass die Nutzenden bei der Interaktion mit der Wallet in Person beobachten werden (z. B. legen die Nutzenden den Personalausweis richtig an das Smartphone an, um diesen zu digitalisieren oder erfassen sie den auf der Webseite des Dienstanbieters angezeigten QR Code korrekt). Um dies zu kompensieren, wurden die Nutzenden entweder gebeten, ihre Handlung in der Kamera des online Meetings vorzuführen, oder diese äußerst detailliert zu beschreiben.

**Abb. 5:**
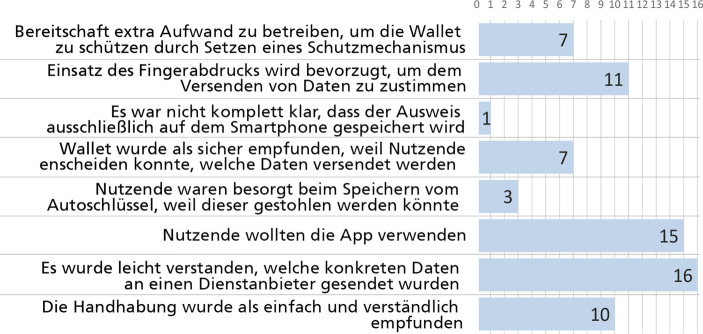
Ergebnisse der Studie aus dem Jahr 2020

Die Studie zeigt immerhin, dass eine große Anzahl der Teilnehmenden (15 von 16) von diesem Konzept überzeugt waren und die Wallet nutzen möchten (siehe Abb. 5). Somit war es möglich die Forschungsfrage 1 positiv zu beantworten (siehe Abschn. 1). Sie sahen darin einen großen Mehrwert für sich selbst, weil es nicht nur die Verwaltungsprozesse vereinfacht, sondern auch den Zugang zu verschiedenen Anwendungsfällen ermöglicht. Dies wurde vor allem deshalb begrüßt, weil das Smartphone als das Gerät gesehen wurde, z. B. (P9): „Das war einfach. Dann muss ich nur noch mein Smartphone dabeihaben. Das habe ich sowieso immer dabei. Aber mein Portemonnaie vergesse ich ab und zu“.

Diese Erkenntnis, dass die Bundesbürger einen digitalen Personalausweis nutzen wollen, wurde in einer Studie von (PwC-Studie 2021) ein Jahr später im Jahr 2021 bestätigt.

Hinsichtlich der zweiten Forschungsfrage (siehe Abschn. 1) war es für die Mehrheit der Teilnehmenden (15 von 16) klar, dass der digitale Personalausweis auf dem Smartphone gespeichert ist. Die Autoren gehen davon aus, dass ein ausreichendes Verständnis des Wallet-Konzepts und seiner Funktionalitäten den Teilnehmenden hilft, das richtige Sicherheitsverhalten im Umgang mit ihren digitalen Identitäten zu wählen.

Die Studie zeigt auch, dass der Identifizierungsprozess gut verstanden wurde. Nicht nur, dass es allen Teilnehmenden (16 von 16) gelang, sich digital zu identifizieren, es war auch für alle Teilnehmenden immer klar, welche persönlichen Daten an welchen Dienst gesendet wurden (siehe Abb. 5). Dies gab ihnen nicht nur das Gefühl, die Kontrolle über den Prozess zu haben, sondern auch, die einzelnen Schritte des Prozesses gut nachvollziehen zu können. Dies wurde nach Angaben der Teilnehmenden auch durch die einfache Handhabung der Wallet unterstützt: 10 von 16 Teilnehmenden bestätigten, dass sie das einfache Design der Wallet schätzten, welches auch leicht zu verstehen war (siehe Abb. 5).

Da diese Anwendung mit persönlichen Daten arbeitet, war es bei der Entwicklung des Konzepts besonders wichtig, den Nutzenden den Eindruck zu vermitteln, dass sie jederzeit die Kontrolle über ihre eigenen Daten haben. Die Ergebnisse der Studie deuten darauf hin, dass die Teilnehmer dies wahrnehmen. So bestätigten die Teilnehmer, dass sie die Tatsache begrüßen, dass die Wallet einen separaten Schutzmechanismus zum Schutz der Daten bietet. Die Studienteilnehmer haben damit nicht nur gezeigt, dass sie die Notwendigkeit des Schutzes ihrer Daten erkannten, sie waren sogar bereit, diesen zusätzlichen Aufwand zu betreiben, um ihre Daten vor unbefugtem Zugriff zu schützen (7 von 16) (siehe Abb. 5). Dabei bevorzugte ein Großteil der Teilnehmenden die Verwendung des Fingerabdrucks zum Schutz der Wallet (11 von 16) (siehe Abb. 5). Somit war es auch möglich Rückschlüsse zur Beantwortung der dritten Forschungsfrage zu ermitteln (siehe Abschn. 1).

Nicht nur, weil ein Schutzmechanismus einzustellen war, sondern auch, weil die Daten erst versendet werden konnten, nachdem die Nutzenden sie gesehen und geprüft (siehe Abb. 4a) und zusätzlich durch Eingabe eines Passworts bestätigt hatten (siehe Abb. 2c), hatten die Teilnehmenden den Eindruck, dass die Wallet sicher war (7 von 16) (siehe Abb. 5). Allerdings gab es noch Bedenken bezüglich der Speicherung eines Schlüssels. Hier gaben 3 von 16 Teilnehmenden an, dass sie sich zu große Sorgen um den Verlust des Autoschlüssels und den damit verbundenen Schaden machen würden (siehe Abb. 5).

### Vertrauen

Da mit dieser Applikation personenbezogene Daten gespeichert werden, war es im Rahmen dieser Studie wesentlich herauszufinden, nicht nur inwieweit Nutzenden das Konzept der Identity Wallet verstehen, sondern ob sie diesem auch vertrauen und glauben, dass dieses Konzept in der Lage ist ihre Daten sicher und privatsphärenfreundlich zu verwalten.

Um das Vertrauen zu ermitteln, wurden die Teilnehmen im Rahmen des offenen Abschlussinterviews gefragt, inwieweit sie dem Konzept vertrauen. Unabhängig davon, welche Antwort (ob ja oder nein) gegeben wurde, sollte eine Begründung geliefert werden.

**Abb. 6:**
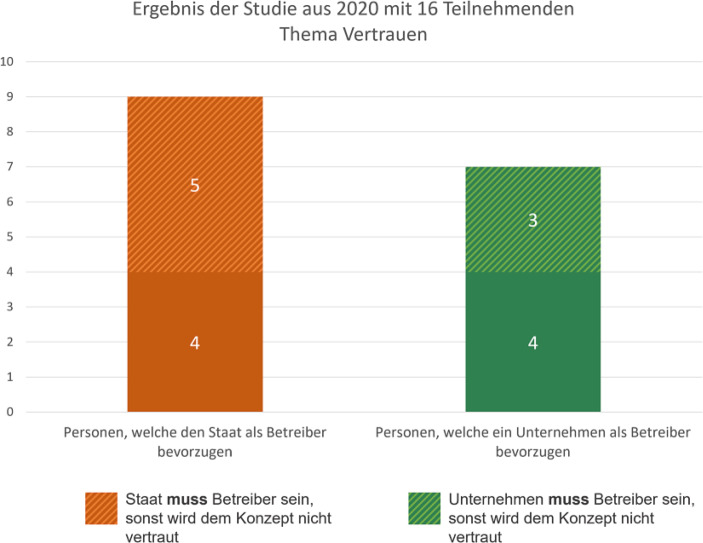
Fokus auf das Thema Vertrauen

Das Ergebnis war, dass die Teilnehmenden grundsätzlich dem Konzept der Wallet vertrauen (16 von 16). Allerdings folgte, ohne den Bedarf einer zusätzlichen Frage, stets die Ergänzung von Seiten der Teilnehmenden, dass ihr Vertrauen stark vom Betreiber der Wallet abhängig sei (siehe Abb. 6). Hier konnten stark unterschiedliche Meinungen identifiziert werden. 9 von 16 Teilnehmenden bevorzugten den Staat als Wallet-Betreiber. Diese Gruppe sah die Rechtfertigung darin, dass der Staat bereits hoheitliche Dokumente zur Verfügung stellt. Daher sahen die Teilnehmenden es nur als sinnvoll an, dass der Staat auch bei dieser Art von Lösung eine bedeutende Rolle spielt. 5 von diesen 9 Personen waren zudem sehr strikt in ihrer Einstellung und sahen den Staat als einzige Option an (siehe Abb. 6). Einer der Teilnehmenden (P16) sagte dazu: „Wenn die Wallet den Personalausweis verwaltet, macht es für mich nur Sinn, wenn der Staat die App betreibt. Ansonsten würde ich der Anwendung nicht vertrauen.“ Andere wiederum gaben an, dass die Unternehmen nur an den Daten interessiert seien, weshalb diese als Betreiber der Wallet nicht in Frage kämen.

Die restlichen 7 von 16 Teilnehmern bevorzugten ein privates Unternehmen als Betreiber der Wallet. Ihre Argumentation war ähnlich, mit dem Unterschied, dass sie den Staat als die Partei sahen, die nur an den Daten interessiert ist. Daher fühlten sie sich wohler, wenn ein privates Unternehmen für die digitale Identität zuständig ist. Auch hier gab es erneut eine Untergruppe von Teilnehmenden (3 von 7), welche nicht kompromissbereit waren und ein privates Unternehmen als einzige Option wahrnahmen.

## Neues Wallet-Konzept

**Abb. 7:**
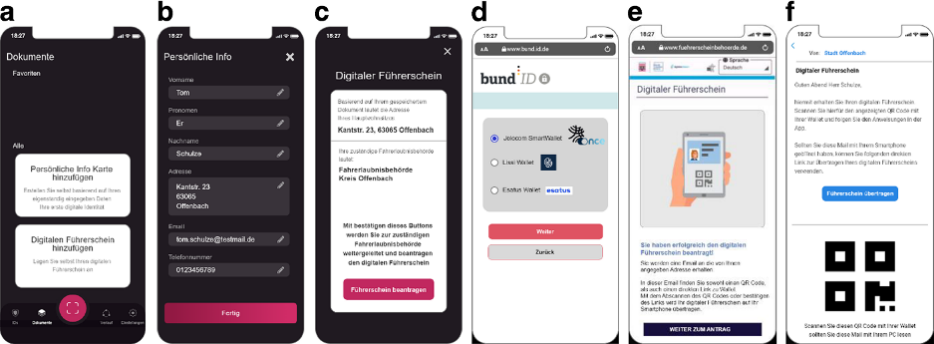
Neues Wallet-Konzept mit Screens zum Anwendungsfall digitaler Führerschein. **a** Übersicht, welche Identitäten in der Wallet gespeichert werden können, **b** Gespeicherte Daten im Rahmen der persönlichen Info-Karte, **c** Hinweis auf die Weiterleitung zur Führerscheinbehörde nach Bestätigung des Buttons, **d** Auswahl des Wallet Anbieters, um sich mit diesem online auszuweisen, **e** Rückmeldung über die erfolgreiche Beantragung des digitalen Führerscheins, **f** Hinweis, dass der digitale Führerschein in die Wallet übertragen werden kann

Da das Wallet Konzept mit zusätzlichen Funktionen erweitert werden sollte, welches sowohl den Einsatz der Onlineausweisfunktion sowie den digitalen Führerschein vorsah, wurde das Konzept basierend auf den Rückmeldungen der Nutzerstudie aus 2020 überarbeitet. Dieses ist in Kooperation mit dem Unternehmen Jolocom^9^ entstanden, welches Betreiber einer SSI Wallet ist. Inspiriert durch ihren Funktionsumfang wurde ein High Fidelity Klick Dummy der Wallet entwickelt (siehe Abb. 7).

Dieser weist folgende Funktionen auf:

1. Bereitstellung von Einleitungsscreens zur Vorstellung des Funktionsumfangs der Wallet
2. Setzen eines Schutzmechanismus zum Entsperren der Wallet
3. Nutzung der Onlineausweisfunktion innerhalb der Wallet
4. Erstellung der Persönlichen Info-Karte (siehe Abb. 7b)
5. Beantragung des Führerscheins
6. Beantragung des mobilen Führerscheins
7. Übertragung des mobilen Führerscheins in die Wallet (siehe Abb. 7f)

Einige der Funktionen werden im Folgenden näher beschrieben.

### Nutzung der Onlineausweisfunktion innerhalb der Wallet

Die Onlineausweisfunktion ist die Funktionalität, welche das Online-Ausweisen mit dem Personalausweis ermöglicht (Bundesministerium des Inneren und für Heimat 2022b). Diese Funktion wird unter anderem von der AusweisApp2 angeboten. Um nicht auf eine andere App zurück greifen zu müssen und dennoch den Personalausweis auf einem hoheitlichen Niveau einsetzen zu können, wurde diese Funktion in das Wallet Konzept integriert.

### Erstellung der persönlichen Info-Karte

Hinter der Persönlichen Info-Karte verbirgt sich die Idee, dass sämtliche Informationen, welche Nutzenden z. B. für die Registrierung benötigen, zusammengetragen werden (siehe Abb. 7b). Dazu gehören Daten wie Name, Anschrift, Telefonnummer, Emailadresse und Geburtsdatum. So können Nutzende mit einem Klick sämtliche erforderlichen Daten übertragen und den Registrierungsprozess nicht nur schneller abschließen, sondern auch verhindern, dass fehlerhafte Daten eingetragen werden (z. B. ein Tippfehler beim Eintragen der Adresse im Antragsformular).

### Beantragung des Führerscheins

Anders als beim Wallet-Konzept im Jahr 2020 erfolgt die Beantragung des Führerscheins ausschließlich über das Smartphone. Dafür erhalten die Nutzenden bereits in der Wallet den Hinweis, dass der Führerschein digitalisiert werden kann (siehe Abb. 7a). Nach dem Klick dieser Fläche, werden die Nutzenden darauf hingewiesen, dass sie für die Beantragung des Führerscheins zur zuständigen Führerscheinbehörde weitergeleitet werden (siehe Abb. 7c). Die zuständige Behörde wird anhand der Anschrift ermittelt, welche die Wallet durch die Persönliche Info-Karte erfasst hat.

Mit Bestätigen des Buttons, werden die Nutzenden zur Webseite der Behörde weitergeleitet. Dort werden die Nutzenden aufgefordert sich online mit dem Personalausweis auszuweisen. Hierbei erhalten die Nutzenden den Hinweis, dass verschiedene Wallets kompatibel mit diesem Ausweisprozess sind (siehe Abb. 7: Neues Wallet-Konzept mit Screens zum Anwendungsfall digitaler Führerschein d). Da in der Jolocom Wallet die Onlineausweisfunktion integriert ist, können die Nutzenden diese App nutzen, um sich digital mit ihrem Personalausweis auszuweisen.

Mit der erfolgreichen Identifikation und dem Abschließen des Antragsprozesses, bekommen die Nutzenden den Hinweis, dass der digitale Führerschein beantragt wurde (siehe Abb. 7e). Sobald dieser zur Verfügung steht, erhalten die Nutzenden eine E‑Mail der Führerscheinbehörde, welche einen Link als auch einen QR Code enthält, um dann den digitalen Führerschein in die Wallet zu übertragen (siehe Abb. 7f).

Mit Bestätigen dieses Links öffnet sich die Wallet. Die Nutzenden werden aufgefordert ihre Führerscheinnummer einzugeben und erhalten dann ihren digitalen Führerschein als Dokument.

## Zweite Nutzerstudie (2022)

Der Ansatz dieser Nutzerstudie gleicht der im Jahr 2020. Auch hier bestand das Ziel darin nicht nur zu ermitteln, ob die Nutzenden das Konzept verstehen, sondern auch inwieweit sie diesem Vertrauen. Zudem hatte diese Studie einen starken Fokus auf den Anwendungsfall „digitaler Führerschein“ und sollte dazu dienen, den Antragsprozess zu verbessern.

Für diese Studie wurden im Sommer 2022 erneut 12 Teilnehmende über die Plattform Testing Time akquiriert. Auch in diesem Fall erhielten sie eine Aufwandsentschädigung. Die Studie nahm pro Teilnehmenden eine Stunde ein.

Auf Grund der anhalten Bedingungen der Pandemie, wurde diese Studie ebenso virtuell durchgeführt. Dafür haben die Teilnehmenden der Studie Zugriff auf den Klick Dummy erhalten und wurden für die Beobachtung ihrer Interaktionen gebeten ihren Bildschirm zu teilen. Um den Klick Dummy evaluieren zu können, erhielten die Teilnehmenden wie im Jahr 2020 Aufgabenstellungen, welche mit dem Prototyp gelöst werden sollten. Um weitere Einzelheiten über ihr Verständnis des Anwendungsfalls zu erfahren, wurden den Teilnehmenden nach jeder Aufgabe Detailfragen gestellt.

Die Aufgaben umfassten:

1. Einrichtung der Wallet und Festlegen der Wallet-PIN
2. Erstellung der Persönlichen Info-Karte
3. Beantragung des physischen Führerscheins mit der Wallet
4. Online-Identifikation mit der integrierten Onlineausweisfunktion der Wallet
5. Zusätzliche Beantragung des digitalen Führerscheins
6. Übertragung des digitalen Führerscheins in die Wallet

Da im Rahmen dieser Studie angenommen wurde, dass die Teilnehmenden über keinen physischen Führerschein verfügen, wurde jedem die Aufgabe gestellt diesen initial zu beantragen. Anschließend war es den Teilnehmenden möglich auch zusätzlich den digitalen Führerschein zu beantragen. Dies sollte ein Szenario von Personen nachstellen, welche zum ersten Mal einen Führerschein beantragen.

Wie auch 2020 wurden im Rahmen der Aufgabenstellungen die Methode Think Aloud angewendet, um sowohl den Handlungen als auch den Gedanken der Teilnehmenden folgen zu können.

Am Ende der Studie wurden dieselben Fragen im Rahmen des Abschlussinterviews wie 2020 gestellt, um wiederum den Gesamteindruck der Wallet und der Anwendungsfälle zu ermitteln.

## Ergebnisse der zweiten Nutzerstudie

Die Ergebnisse dieser Studie bestätigten erneut den Hinweis, dass Nutzende bereit sind das Konzept einer Identity Wallet einzusetzen zu wollen (12 von 12). Nahezu allen Teilnehmenden (11 von 12) ist es ohne Hilfestellung gelungen den Beantragungsprozess des Führerscheins erfolgreich abzuschließen und sie begrüßten es, dass dieser Prozess digital angeboten wird.

Am Ende der Studie wurde erneut die Frage hinsichtlich des Vertrauens in solch ein Konzept gestellt. Wie auch 2020 sollten die Teilnehmenden der Studie sämtliche Antworten begründen.

Auch 2022 äußerten Teilnehmenden, dass sie dem Konzept grundsätzlich vertrauen (12 von 12). Anders als 2020 hatten die Teilnehmenden jedoch nicht eigenständig ergänzenden Äußerung hinsichtlich ihres idealen Betreibermodells getätigt. Diese Rückmeldung wurde daher durch eine zusätzliche Frage ermittelt.

**Abb. 8:**
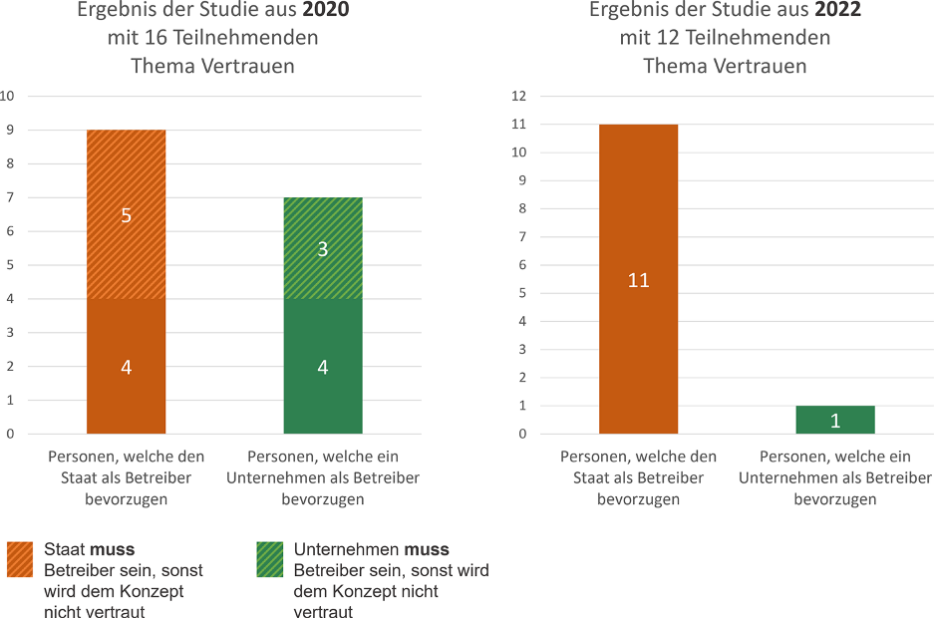
Gegenüberstellung der Ergebnisse der Studie hinsichtlich des Themas Vertrauen im Jahr 2020 und 2022

Das Ergebnis ist, dass sich das Mehrheitsverhältnis und die Meinungsbilder zwischen den Gruppierungen, welche den Staat als Betreiber wollten und denjenigen, die ein Unternehmen als Betreiber wünschten, im Vergleich zum Jahr 2020 wesentlich geändert haben (siehe Abb. 8).

Nun geben eine deutliche Mehrheit an (11 von 12), dass sie den Staat als Betreiber der Wallet wünschen. Die Begründung blieb dieselbe wie auch zwei Jahr zuvor. Da hoheitliche Dokumente in der Wallet eingesetzt werden, welche vom Staat bereitgestellt werden, sollte auch der Staat im dem Betreibermodell der Wallet eingebunden werden. Nur eine Person bevorzugte ein privates Unternehmen als Betreiber der Wallet. Die Begründung war eine andere als im Jahr 2020. Statt das Sorge bekundet wurde, dass der Staat an den persönlichen Daten interessiert sei und daher ein privates Unternehmen besser geeignet wäre, wurde im Jahr 2022 die Entscheidung in der Form begründet, dass der Staat als nicht kompetent eingeschätzt wird. Ein privates Unternehmen sei besser aufgestellt und hat in der Vergangenheit, durch z. B. das Anbieten einer Apple Wallet bewiesen, dass sie in der Lage wären, solche Applikation korrekt umzusetzen.

## Diskussion – Vergleich der Ergebnisse

In dem Zeitraum, in dem die Studie 2020 durchgeführt wurde, wurde in Deutschland die sogenannte Corona-Warn-App veröffentlicht, die eine datenschutzfreundliche Kontaktverfolgung zur anonymen Identifizierung von Infektionsketten ermöglichen sollte. Da es sich um die erste staatliche Anwendung handelte, die in dieser Form veröffentlicht wurde, wurde die Corona-Warn-App in den deutschen Medien stark diskutiert. Dabei wurde häufig die Frage aufgeworfen, inwieweit die App tatsächlich datenschutzfreundlich sei und nicht einer staatlichen Überwachung diene (The Guardian 2020; Zeit Online 2020). Da während der Studie im Jahr 2020 von den Teilnehmenden häufig Hinweise auf die Corona-Warn-App geäußert wurden und diese App stark in den Medien vertreten war, kann vermutet werden, dass die Teilnehmenden stark für das Thema Sicherheit und Datenschutz sensibilisiert waren. Zudem ist es möglich, dass die Diskussion um die Corona-Warn-App einen wesentlichen Einfluss auf die Entscheidung über den Wallet-Betreiber hatte, was zur Bildung dieser beiden Gruppen geführt haben könnte (Xing et al. 2021).

Diese Vermutung wird dadurch bestärkt, da sich im Jahr 2022 das Meinungsbild hinsichtlich des Betreibermodells der Wallet komplett gewandelt hat. Zu dieser Zeit war die Corona-Warn-App lange nicht mehr so stark in den Medien diskutiert worden (Google Trends 2023). Im Jahr 2022 erfolgten von Seiten der Teilnehmenden ebenso keinerlei Hinweise mehr auf die Corona-Warn App.

Dies lässt vermuten, dass durch die fehlende Diskussion in den Medien und starke Kritik an dem Staat hinsichtlich technologischer Angebote die Nutzenden nicht mehr so stark kritisch dem Staat gegenüber eingestellt waren.

Zudem war zu beobachten, dass die Teilnehmenden der Studie 2020 weitaus mehr grundsätzliche Bedenken hinsichtlich des Betreibermodells geäußert hatten als die Teilnehmenden im Jahr 2022. 2020 fielen Äußerungen wie „Open Source“, „Evaluierung von Dritten“ oder „Chaos Computer Club“. Dies ist ebenfalls eine Parallele zur Corona- Warn-App, weil diese Worte häufig zusammen mit der App in den Medien erwähnt wurden. Im Jahr 2022 hat nicht einer der Teilnehmenden eines dieser Worte erwähnt. Dies lässt vermuten, dass allein der Kontext „Umgang mit persönlichen Daten“ bei Nutzenden nicht dazu führt, sich stärker Gedanken hinsichtlich Sicherheit oder Privatsphäre zu machen.

Grundsätzlich lässt sich durch dieses Ergebnis vermuten, dass starke Diskussionen in den Medien hinsichtlich eines Themas, welches für die Nutzenden interessant ist, einen Einfluss auf die Ergebnisse von Studien nimmt und bei der Durchführung berücksichtig werden sollte.

## Einschränkungen

Sowohl für die Studie im Jahr 2020 als auch für die Studie im Jahr 2022 wurden qualitative Umfragen durchgeführt. Da die Anzahl der Studienteilnehmenden mit jeweils 16 und 12 Personen relativ gering ist, zeigen die aktuellen Ergebnisse Tendenzen auf. Diesen können durch quantitative Studien mit einer größeren Anzahl an Studienteilnehmenden in einer zukünftigen Untersuchung untermauert werden.

Zudem wurden die Studien in diesem Beitrag in einem Umfeld in Deutschland durchgeführt. Eine Erweiterung des Kreises der Teilnehmenden auf weitere Nationalitäten könnten Rückschlüsse dazu liefern, inwieweit andere Nationen eine ähnliche oder differente Präferenz zu dem Betreiber einer Wallet im Vergleich zu Deutschland aufzeigen.

Darüber hinaus ist anzumerken, dass beide Studien online durchgeführt wurden. Damit war es nicht möglich die Nutzenden direkt bei der Interaktion mit der Wallet zu beobachten, um unter anderem damit zu erfahren, inwieweit die Nutzenden die Interaktion mit einer Gegenstelle verstehen.

Da in beiden Studien Klick Dummies getestet wurden, fehlen ebenso Rückmeldung zu echten Interaktionen einer Applikation. Dies soll nachgeholt werden, sobald ein implementierter Demonstrator der Wallet App mit umfangreichem Funktionsumfang existiert.

## Zusammenfassung und Ausblick

In diesem Beitrag wurde ein App-Konzept mit mehreren Identitäten (Wallet) vorgestellt und mehrere Nutzerstudien durchgeführt, um die Akzeptanz für die Nutzung einer digitalen Wallet und das Verständnis des Identifizierungsprozesses zu ermitteln. Die Ergebnisse deuten darauf hin, dass das Konzept gut verstanden wurde, einschließlich des Prozesses der Digitalisierung von Ausweisdokumente und des Identifikationsprozesses. Darüber hinaus wurde die Wallet als sicher und einfach zu benutzen angesehen, und die Nutzenden gaben an, dass sie das Gefühl haben, die Kontrolle über ihre Daten zu haben.

Da die Wallet allein reicht, um sie bei unterschiedlichsten Diensten einzusetzen, erkannten die Teilnehmenden sofort den Mehrwert der App und zeigten eine große Bereitschaft, sie zu nutzen. Auch dem Konzept wurde vertraut, wobei dies bei den Teilnehmenden bei der Studie im Jahr 2020 davon abhing, ob der Betreiber der Wallet der Staat oder ein privates Unternehmen war. Hier haben sich in etwas zwei gleich große Gruppierungen gebildet.

Eine weitere Studie mit Nutzenden wurde 2022 zur erneuten Evaluierung des Wallet-Konzepts durchgeführt. Die Ergebnisse dieser Studie wiesen darauf hin, dass die Teilnehmenden der Studie weiterhin interessiert sind die Wallet einzusetzen. Hinsichtlich des Thema Vertrauens hat sich das Bild deutlich geändert. Die Nutzenden vertrauen dem Wallet-Konzept weiterhin, aber statt der zwei gleich großen Gruppen, zeigten die Ergebnisse auf, dass nur noch eine Person sich ein privates Unternehmen als Betreiber wünscht und die übrigen den Staat als Betreiber bevorzugen.

Da beide Studien online durchgeführt wurden, ist eine Durchführung einer Studie in Präsenzform in weiteren Untersuchungen geplant. Sie würde dabei unterstützen die tatsächlichen physischen Interaktionen der Nutzenden mit der Wallet und der Gegenstelle zu beobachten und damit mehr Rückschlüsse zum Verständnis des Einsatzes einer Wallet liefern. Zudem ist eine Wiederholung der Nutzerstudie aus 2020 geplant, um erneut das Meinungsbild zur Wahl des Betreibers der Wallet zu ermitteln. Dies dient der Untersuchung, ob ggf. das Design der Mockups aus 2022 Einfluss auf die Entscheidung des favorisierten Betreibers der Wallet genommen hat. Zu guter Letzt bliebt die Untersuchung der Frage, inwieweit ein gutes Verständnis des Konzepts der Wallet und ihrer Funktionalitäten mit einem angemessenen Sicherheitsverhalten beim Umgang mit digitalen Identitäten verbunden ein Thema für künftige Arbeiten.

## Supplementary Information

Leitfaden der Nutzerstudie – 2020

Leitfaden der Nutzerstudie – 2022

## Footnotes

1 https://developers.facebook.com/docs/facebook-login/?locale=en_US (Zugriff 25.09.2022).

2 https://developers.google.com/identity/sign-in/web/sign (Zugriff 25.09.2022).

3 https://www.ausweisapp.bund.de/home (Zugriff 25.09.2022).

4 Dieses Konzept ist im Jahr 2020 entstanden und ein eigenständiger Entwurf einer Wallet Applikation. Es steht nicht in Verbindung mit dem Projekt „ID Wallet-App“ der deutschen Bundesregierung (https://www.bundesregierung.de/breg-de/suche/e-id-1962112) (Zugriff: 25.09.2022).

5 https://www.testingtime.com/en/ (Zugriff: 25.09.2022).

6 Ein Secure Element ist ein hardwarebasierter Chip auf mobilen Endgeräten, welcher Schutz gegen unautorisierten Zugriff ermöglicht.

7 Beispielsweise bei einer App zu App Kommunikation zwischen der App des Dienstanbieters und der Wallet App.

8 Beispielsweise bei einer Web zu App Kommunikation zwischen der Website eines Dienstanbieters mit dargestellten QR Code und der Wallet App.

9 https://jolocom.io/de/ (Zugriff 25.09.2022).
