# Supplementary material for: The change of trust into a digital identity? – Insights into a user study
Source: HMD Prax Wirtsch Inform. 2023 Mar 2;60(2):322–43. [Article in German] doi: 10.1365/s40702-023-00951-7 (PMC9980846; doi:10.1365/s40702-023-00951-7)
Supplement: Supplementary file 1 — Leitfaden der Nutzerstudie – 2020 [file 40702_2023_951_MOESM1_ESM.docx]

# Leitfaden der Nutzerstudie - 2020

## ***App einrichten:***

„Ihre erste Aufgabe besteht darin die App einzurichten. Starten Sie nun also die ID Wallet App und folgen Sie den Anweisungen!“

**Fragen:**

1. *Sie haben nun Ihre App eingerichtet. Wurde für Sie deutlich, was der Funktionsumfang der eingerichteten App ist?*
2. *Wurde für Sie deutlich, wofür die PIN / PW oder Fingerprint gesetzt werden soll?*
3. *Wie ist ihre Einstellung zur Verwendung des Fingerabdrucks z.B. zur Authentisierung?*
4. *Bevorzugen Sie es den Sperrmechanismus vom Smartphone zu übernehmen oder ein neues Passwort zu setzen?*

## ***Personalausweis digitalisieren:***

„Sie haben Ihre ID Wallet eingerichtet. Weil Sie sich bei einem anderen Service ausweisen wollen, benötigen Sie nun eine Digitale Identität.“

**Fragen**

1. *Können Sie bitte kurz in Ihren eigenen Worten wiedergeben, was sie gerade gemacht haben?*
2. *Sie haben nun einen digitalen Personalausweis: Welchen Eindruck vermittelt Ihnen die App, wo ihre Identität gespeichert ist?*
3. *Was ist Ihr Eindruck, was Sie nun mit diesem digitalen Ausweis machen können?*
4. *Was erhoffen Sie sich mit dem digitalen Ausweis machen zu können?*
5. *Welche Daten wurden nun durch den Vorgang erfasst?*

## **Führerschein erstellen (Web zu App Kommunikation)**

„Sie haben den Service entdeckt, dass Sie nicht nur den Personalausweis, sondern auch den Führerschein digital auf den Smartphone speichern können. Sie rufen hierfür den Service über Ihrem PC in einem Webbrowser auf. Ihre Aufgabe besteht nun darin den digitalen Führerschein anzulegen.“

**Fragen**

1. *Können Sie bitte kurz in Ihren eigenen Worten wiedergeben, was sie gerade gemacht haben?*
2. *Für die Beantragung des digitalen Führerscheins haben Sie sich zuerst identifizieren müssen. War für Sie hier transparent, welche Daten die Bund.de Webseite für eine Identifizierung von Ihnen benötigt?*
3. *Die Identifizierung war erfolgreich. Die Bund.de Webseite hat anscheinend die geforderten Daten erhalten. Wurde deutlich, welche Daten Sie der Bund.de Webseite zugeschickt haben?*
4. *Welchen Eindruck hatten Sie womit Sie sich ausgewiesen habe?*
5. *Welcher Eindruck wurde Ihnen vermittelt, wie Sie den digitalen Führerschein bekommen haben?*
6. *Im Prozess mussten Sie zwei Mal einen QR-Code einscannen. Welcher Eindruck wurde Ihnen vermittelt wofür diese beiden QR-Code verwendet wurden (Worin sehen Sie einen Unterschied?)*

## ***Registrierung bei der Bibliotheks App (*App zu App Kommunikation*):***

„Neben einem digitalen Personalausweis, können Sie auch Daten händisch eingeben und Ihrer ID Wallet speichern.

[Zeigen, was bereits in der Wallet gespeichert ist]

Für Ihrer Arbeit benötigt Sie ein Buch als Grundlage für Ihre Recherchen und haben eine Bibliothek entdeckt, bei denen Sie auch einen digitalen Büchereiausweis erstellen und in Ihrer Wallet speichern können. Ihre Aufgaben besteht nun darin diesen Ausweis anzulegen.“

**Fragen**

1. *Können Sie bitte kurz in Ihren eigenen Worten wiedergeben, was sie gerade gemacht haben?*
2. *Für die Identifizierung will ein Anbieter ja in der Regel ein paar Daten von Ihnen haben. War für Sie hier transparent, welche Daten die Bibliothek für eine Identifizierung von Ihnen benötigt?*
3. *Die Identifizierung war erfolgreich. Die Bibliothek hat anscheinend die geforderten Daten erhalten. Wurde deutlich, welche Daten Sie der Bibliothek zugeschickt haben?*
4. *Die Daten, welche Sie verschicken konnten, wurde in zwei Gruppen eingeteilt. Worin sehen Sie den Unterschied (verifiziert und bearbeitbar)?*
5. *[Wenn die Person sich den Dienst näher angesehen hat] Was war ihre Motivation mehr zu dem Dienst zu erfahren?*
6. *Wurde deutlich, wie die Bibliothek die Daten erhalten hat?*

1. *Hatten Sie bedenken, diese Daten / Ihre Daten an den Service zu senden?*
2. *Was würde helfen die Bedenken zu vertreiben?*
3. *Welcher Eindruck wurde Ihnen vermittelt, wie Sie den digitalen Bibliotheksausweis bekommen haben?*

## ***Fahrzeugschlüssel speichern (*App zu App Kommunikation*)***

„Sie sind im Urlaub und haben dort ein Fahrzeug gemietet, welches Sie bereits gebucht haben. Der Dienst bietet Ihnen nun einen Service an, welche Sie nicht mehr dazu zwingt ihren Fahrzeugschlüssel dabei zu haben.  Ihre Aufgabe besteht nun darin diesen neuen Service zu testen.“

**Fragen**

1. *Können Sie bitte kurz in Ihren eigenen Worten wiedergeben, was sie gerade gemacht haben?*
2. *Ist für Sie deutlich geworden, wo der Schlüssel gespeichert wurde?*
3. *Wie würden Sie jetzt diesen Schlüssel verwenden?*

## **Abschluss Interview:**

1. *Was ist ihr Gesamteindruck einer solchen Anwendung?*
2. *Nutzen Sie im realen Leben ähnliche Anwendungen?*
3. *Wenn ja, welche?*
4. *Wenn nein, warum nicht?*
5. *Würden Sie im realen Leben diese Anwendung nutzen?*
6. *Wenn “ja”: Warum? Worin sehen Sie die Vorteile?*
7. *Wenn “nein”: Warum nicht?*
8. *Wären Sie bereit einer solchen Anwendung Vertrauen zu schenken? Was würde Ihre Bereitschaft beeinflussen?*
9. *Unabhängig, ob jetzt Sie die Anwendung nutzen würden: Worin sehen Sie den Vorteil in der Nutzung einer solchen App?*
10. *Wie stehen Sie zu der Idee einer auf ihrem Smartphone gespeicherten Identität /*
11. *Und wie dazu, dass Schlüssel und ID gemeinsam gespeichert werden?*
12. *Sie habe hier die Anwendung sowohl über eine Webseite als auch über eine App interagierend getestet. Welche Variante bevorzugen Sie?*
13. *Bevorzugen Sie im allgemeinen Web oder App Anwendungen?*
14. *Welche Anwendungen würde Sie im allgemein oder im Besonderen dazu motivieren die ID Wallet dafür zu verwenden? (Gibt es eine Anwendung, wo Sie selber den Bedarf sehen dafür die ID Wallet verwenden zu wollen)*
15. *Ich möchte Ihnen zum Abschluss noch ein paar Fragen zu ihrem allgemeinen Nutzungsverhalten stellen:*
16. *Nutzen Sie vermehrt die Apps, die auf ihrem Gerät installiert sind, oder installieren Sie öfters Apps über Plattformen wie Google Play oder den Apple Playstore?*
