# Supplementary material for: The change of trust into a digital identity? – Insights into a user study
Source: HMD Prax Wirtsch Inform. 2023 Mar 2;60(2):322–43. [Article in German] doi: 10.1365/s40702-023-00951-7 (PMC9980846; doi:10.1365/s40702-023-00951-7)
Supplement: Supplementary file 2 — Leitfaden der Nutzerstudie – 2022 [file 40702_2023_951_MOESM2_ESM.docx]

# Leitfaden der Nutzerstudie - 2022

## ***App einrichten:***

„Ihre erste Aufgabe besteht darin die App einzurichten. Starten Sie nun SmartWallet App und folgen Sie den Anweisungen!“

**Fragen:**

1. [Optional: Nachdem die Einleitung vorgelesen und durchgeklickt wurde]
   *Wenn Sie die App z.B. Zuhause allein eingerichtet hätte, würden Sie dann auch die Einleitung lesen?*
2. [Optional: Person hat die Einleitung übersprungen] *Wieso haben Sie die Einleitung übersprungen?*
3. *Sie haben nun Ihre App eingerichtet. Können Sie in Ihren eigenen Worten wiedergeben, was der vorgestellte Funktionsumfang der App ist?*
4. *Was ist ihr Eindruck. Welchen Zweck hatte das Festlegen einer PIN?*
5. *Wie finden Sie es, dass eine PIN gesetzt werden musste?*
6. *Was ist ihr Eindruck, was Sie mit der Online Ausweisfunktion machen können?*
7. *Wie stellen Sie sich den Ausweisvorgang mit der Online Ausweisfunktion vor?*

## ***Erstellen der persönlichen Info Karte:***

„Dann kommen wir zur nächsten Aufgabe. Ihre Aufgabe ist nun Ihre erste persönliche Identitätskarte anzulegen.“

**Fragen:**

1. *Was ist ihr Eindruck, was Sie mit der persönlichen Info Karte machen können?*
2. *Was ist Ihre Vorstellung, wie Sie die persönliche Info Karte einsetzen werden?*
3. *Würden Sie sich in Ihrem echten Leben eine persönliche Info Karte erstellen? Wieso?*
4. *Was ist Ihr Eindruck, wie Sie Änderungen innerhalb der persönlichen Info Karte vornehmen können?*
5. *Basierend auf den bisherigen Eindruck, würden Sie die Wallet verwenden wollen? Wieso?*

## ***Digitaler Führerschein und eID***

„Wir kommen nun zur nächsten Aufgabe und hier möchte ich Ihnen vorab ein paar Informationen zukommen lassen. Sie haben nun festgestellt, dass Sie in Ihrer Wallet auch den digitalen Führerschein speichern können. Wichtig ist aber zu wissen, dass Sie noch gar keinen physischen Führerschein besitzen. Sie haben sich aber bereits bei einer Fahrschule angemeldet, Ihre Prüfungen sind aber noch ausstehend. Sie möchten jetzt nun den physischen Führerschein für die Fahrerlaubnisklasse B beantragen und damit auch gleichzeitig den digitalen Führerschein. Ihre Aufgabe ist nun die Beantragung aus der Wallet zu starten.

Bei dieser Aufgabe werde ich Sie auch darum bitten Ihre Verhaltensweisen zu beschreiben. Ich werde Sie an der notwendigen Stelle darauf aufmerksam machen“

**Fragen:**

1. *Können Sie kurz mit eigenen Worten so präzise wie möglich zusammenfassen, was Sie gerade gemacht haben?*
2. *Was ist ihr Gesamteindruck dieses Antrags?*
3. *Für die Beantragung des digitalen Führerscheins haben Sie sich identifizieren müssen. War für Sie hier transparent, welche Daten die Webseite für eine Identifizierung von Ihnen benötigt?*
4. *Wurde es für sie deutlich, welche konkreten Daten Sie versendet haben?*
5. *Welchen Eindruck hatten Sie womit Sie sich ausgewiesen habe?*
6. *Welchen Eindruck hatten Sie, wohin Sie die Daten versenden?*
7. *Welcher Eindruck wurde Ihnen vermittelt, wie Sie den digitalen Führerschein bekommen haben?*
8. *Kennen Sie das Nutzerkonto?*
9. *[Falls ja] Haben Sie bereits eines mal angelegt?*
10. *[Falls ja] Haben Sie bereits einmal das Nutzerkonto verwendet?*
11. *Wie ist Ihre Vorstellung den digitalen Führerschein einzusetzen?*
12. *Hat Sie Ihre Einstellung zum Einsatz der Wallet geändert? Wieso?*

## ***Favorisieren der mDL***

„Damit kommen wir zur letzten Aufgabe. Markieren Sie den Führerschein nun als Favoriten.“

**Fragen:**

1. *Worin sehen Sie den Zweck in dieser Funktion?*
2. *Was halten Sie von dieser Funktion?*

## **Abschluss Interview:**

1. *Was ist Ihr Gesamteindruck einer solchen Anwendung?*
2. *Nutzen Sie im realen Leben ähnliche Anwendungen?*
3. *Wenn ja, welche?*
4. *Wenn nein, warum nicht?*
5. *[Optional, abhängig davon, ob bereits gesagt wurde, die Wallet nutzen zu wollen]
   Worin sehen Sie die Vorteile solch eine Anwendung zu nutzen?*
6. *Wären Sie bereit einer solchen Anwendung Vertrauen zu schenken? Was würde Ihre Bereitschaft beeinflussen?*
7. *Wer sollte diese Anwendung anbieten? Wieso?*
8. *[Optional, falls gesagt wurde, dass der Anwendung nicht vertraut wird]
   Was fehlt Ihnen konkret, damit Sie der Anwendung vertrauen?*
9. *Nun folgendes Gedankenexperiment. Sie haben den Hinweis erhalten, dass zur Digitalisierung Ihres Führerscheins mehrere Anwendungen zur Verfügung stehen. Sie haben sich diese Anwendungen angesehen und getestet. Welche Faktoren sind für Sie relevant, wenn Sie beschließen, welcher Anwendung Sie vertrauen und damit auch nutzen wollen.*
10. *Welche Anwendungen würde Sie im allgemein oder im Besonderen dazu motivieren die Wallet zu verwenden?*
11. *Was ist Ihr beruflicher Hintergrund?*
12. *Wie alt sind Sie?*
